# Optimal urethral catheter removal time after robotic radical prostatectomy: a systematic review of the current evidence

**DOI:** 10.3389/fsurg.2025.1731485

**Published:** 2026-01-15

**Authors:** Panagiotis Mourmouris, Nikolaos Kostakopoulos, Omer Burak Argun, Ioannis Georgopoulos, Vasillios Klapsis, Nikolaos Pisiotis, Ioannis Salmas, Tunkut Doganca, Sotirios Charamoglis

**Affiliations:** 1 Urology Department, Metropolitan Hospital, Piraeus, Greece; 2 Urology Department, Metropolitan General Hospital, Athens, Greece; 3 Health Services of Vocational School, Medical Laboratory Techniques, Istanbul Kent University, Istanbul, Türkiye; 4 Department of Urology, Acibadem Taksim Hospital, Istanbul, Türkiye

**Keywords:** catheter, complications, prostate cancer, robotic radical prostatectomy, surgery

## Abstract

**Background:**

Robotic Radical Prostatectomy has become the dominant surgical approach for localized prostate cancer, offering offers many advantages in postoperative recovery and quality of life. Despite these advances, the standard duration of urethral catheterization- typically 7 days- has remained largely unchanged.

**Objective:**

To systematically evaluate the feasibility and safety of early urethral catheter removal after robotic radical prostatectomy and to identify the optimal timing for catheter removal.

**Methods:**

A systematic review was conducted according to PRISMA guidelines. PubMed, Web of Science, Cochrane Library, Google Scholar and Scopus databases were searched from inception to August 2025. Case reports, non robotic studies and non English publications were excluded Study quality was assessed using the Newcastle-Ottawa Scale for non randomized studies and the Jadad scale for randomized controlled trials.

**Results:**

Thirteen studies involving 4.055 patients met inclusion criteria, including three randomized controlled trials. Early catheter removal was variably defined, most commonly between 1 and 4 post operative days. Across studies early removal was not associated with increased rates of anastomotic leakage, urethral stricture or bladder neck contracture. Continence recovery seams to be occur earlier with early removal although higher short term urinary retention rates were reported. Overall complications and readmission rates were low. Study quality was acceptable despite the limited evidence from high quality randomized studies.

**Conclusions:**

Early catheter removal after robotic radical prostatectomy appears both safe and feasible in appropriate selected patients and may accelerate continence recovery without compromising long-term outcomes. Catheter removal on postoperative days 3-4 appears to offer the most favora.

## Introduction

Prostate cancer (PCa) is currently the second most prevalent cancer in men with approximately 1.5 million new cases worldwide in 2020 (1). Its management has been revolutionized by the use of the robotic platform which had rapidly increased to a stunning 85% in 2012 in the USA (2). Robotic radical prostatectomy (RRP) offers several advantages over open prostatectomy due to its minimally invasive nature including reduced pain and discomfort, faster recovery and improved quality of life -benefits that may be further enhanced by early removal of the indwelling folley catheter (3). Reports indicate that in nearly 50% of patients undergoing radical prostatectomy, catheter may cause more pain and discomfort than the incision itself (4).

Despite the potential benefits that early catheter removal provides, the standard of care of 7 days doesn't seem to change even in high volume centers (3). However, some surgeons have questioned this standard of care, removed the catheter earlier and published their results. The basic concerns of anastomosis leakage and urethral stenosis have been addressed in these studies. This is the first systematic review to evaluate the feasibility and safety of early catheter removal with primary goal of determining the optimal timing of the catheter removal after robotic radical prostatectomy.

## Material and methods

### Design and inclusion criteria

Our systematic review was performed according to PRISMA guidelines (5). For this study Institutional Review Board approval was not required. We included in our systematic review: original prospective or retrospective studies, randomized or non-randomized, published in peer reviewed journal, having at least an abstract, in English language, from inception until 2025 whereas we excluded, case reports and case series, comments as well as studies not reporting robotic cases.

### Search strategy

A systematic search of available literature was conducted in August 2025, in PubMed, Web of Science, Cochrane library, Google scholar and Scopus databases. The research question structured according to PICO criteria, focused on men with localized prostate cancer (P), who underwent robotic radical prostatectomy (I), with comparison of different indwelling catheter removal times (C), to evaluate functional outcomes for patients (O).

A combination of related keywords waw used for the search: (Robotic prostatectomy) AND ((Catheter removal time) OR (indwelling catheter) OR (optimal catheter) OR (optimal catheter removal time)). Title screening was independently performed by two authors manually (NK & PM) and consensus among all the authors resolved any discrepancies. After duplicates were removed and non-English studies were excluded, relevant studies were assessed for eligibility, by being subjected to a full-text review, before being included in the systematic review.

### Data extraction and quality assessment

Data extraction was performed using a standardized form shared by both reviewers. Baseline preoperative characteristics of the patients were recorded and also the relevant intra and post operative information were extracted. Complications were graded according to the Clavien-Dindo system (6). The quality of the non-randomized studies was assessed with the NewCastle-Ottawa Quality Assessment Tool(NOS) (7), with a total score 5 or less to be considered as low quality, a score of 6–7 intermediate and a score of 8 and above was considered as high quality. For randomized controlled trials we the Jadad Scale (8) was used.

## Results

**Figure 1:**
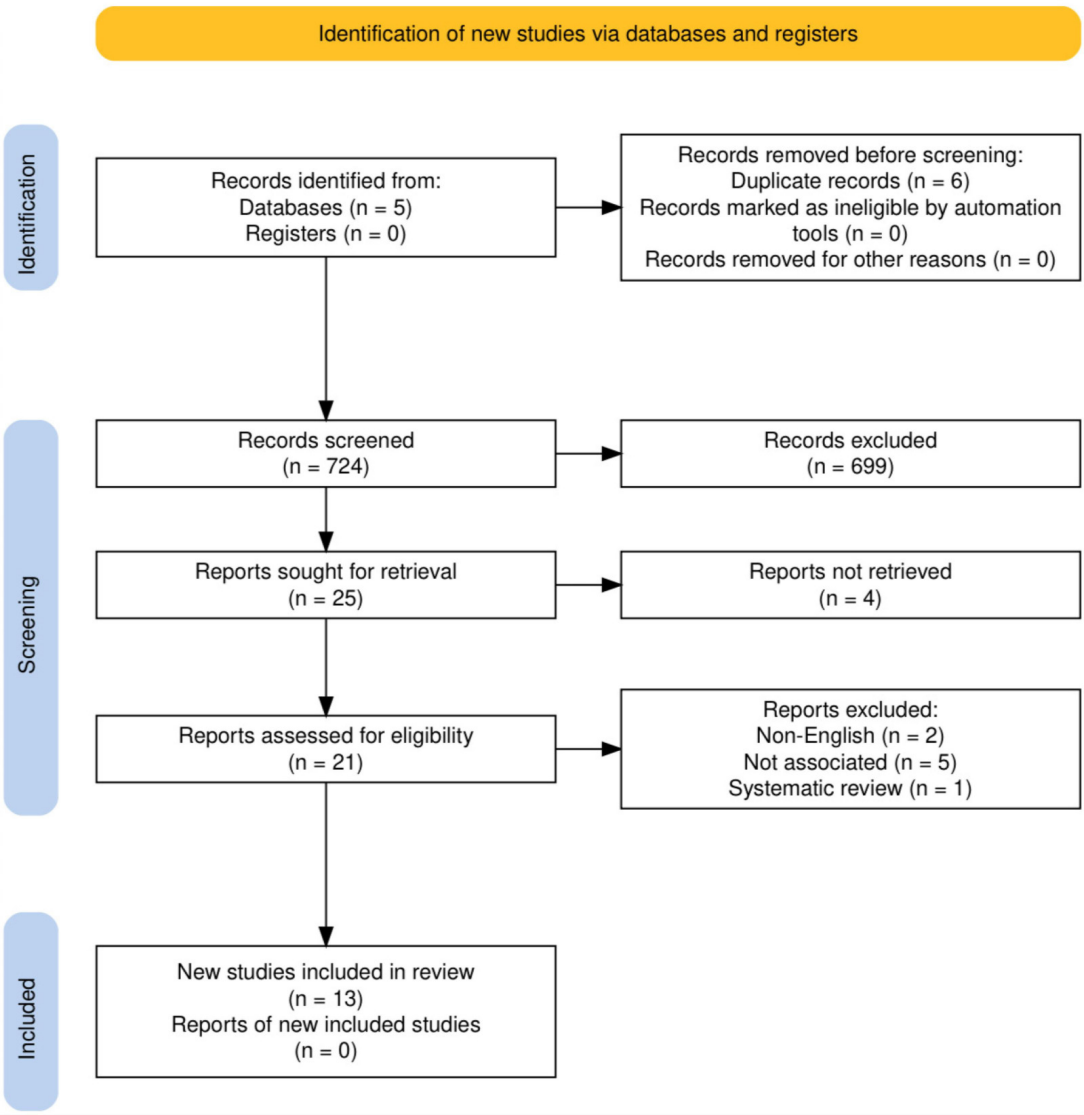
PRISMA flowchart.

Our search yielded 724 papers, of which 699 were excluded after reviewing titles and abstracts. Of the remaining 33 articles 6 duplicates and 2 non-English studies were excluded, leaving 25 articles for full-text analysis. 13 studies met the inclusion criteria and were finally included in our study. The flowchart of our search is presented in Figure 1.

Among the studies included, there were three randomized control trials (RCTs), five nonrandomized prospective trials and the remaining 6 were retrospective. A total of 4,055 patients were enrolled in these trials. The nonrandomized studies scored more than 7 in the NOS score whereas the two RCT scored 1 and 2 in the Jadad scale, finding suggesting that the overall quality of included studies was acceptable. Quality assessment of the studies that were included in our analysis is shown in Table 1.

**Table 1:** Patients basic characteristics and quality assessment of included studies.

Study	Type	Patients (No)	Age (years)	Prostate volume (cm^3^)	IPSS (mean)	High risk(D’ Amico)/ Locally advanced disease	Quality Assessment
Harke et al. (2020)	RCT	198	65 (TC & SPT2) 66 (SPT 5)	44 (TC), 35 (SPT5), 43 (SPT2)	4 (TC) 6 (SPT 2 &5)	12 (TC), 14 (SPT5), 8 (SPT2) Gleason 8–10	2
Lista et al. (2018)	RCT	146	Median 63 (TC 3) 64 (TC 5)	Median 43,5(TC3) 44 (TC 5)	Median 6 (TC3) 8 (TC 5)	cT3 4% (TC3) vs. 2,7% (TC5)	2
Prasad et al. (2014)	RCT	58	57.7 ± 8.6 (TC) 60.0 ± 6.4 (SPT)		6.8 ± 5.7 (TC) 7.5 ± 6.5 (SPT)	Gleason 7 or greater 17 (TC) vs. 8 (SPT) *p* = 0,02	1
Lenart et al. (2024)	Prospective	132	67,4 ± 6,5 (>14) 61,5 ± 7,3 (<14)	57,1 ± 20,2 (>14) 48,9 ± 11,9 (<14)		pT: 3,2 ± 1 (>14) 3,1 ± 1,1 (<14)	7
Paludo et al.	Prospective	21	62 (median)			median Gleason score 3 + 4	7
Gratzke et al.	Prospective	74	62(pod 2), 65(pod 6) (median)	35 (pod 2), 40 (pod 6) (median)	4	2% (pod 2) 11% (pod 6) High risk	8
Brasetti et al.	Prospective	138	62.2 ± 5.8 (mean ± SD)	43.7 ± 14.5 (mean ± SD)	7 ± 6.1	26% (>pT3a) 14% high risk	8
Hao et al.	Retrospective	432	66,5 (median)	35 (median)	7	28,7% Gleason 8–10	7
Taylor et al.	Retrospective	115	64.47 ± 7.05 (SPT 6/ TUC 1) 62.94 ± 7.44 (SPT 6 only/no TUC)			24,35% (14 & 14) Gleason score 8–10	7
Lenart 2 et al. (2019)	Retrospective	425	65,19 (mean)	52,01 (mean)		16,6% Gleason 8–10	7
Alnazari et al.	Retrospective	740	60,44 (mean)	50,22 (mean)	7,6	0,8% (cT3a)	8
Develtere et al.	Retrospective	369	67 (median)	40 (mean)		22% (cT3a) 26% (High risk)	7
Khemees al. (2013)	Retrospective	1026	59.8 ± 7 (AUR patients) 60.1 ± 7 (no AUR patients) (mean ± SD)	60.3 ± 29.2 (AUR patients) 55.7 ± 22.2 (no AUR patients) (mean ± SD)			7

SPT, suprapubic tube; TUC, transurethral catheter; IPSS, International Prostate Symptom Score, Quality Score according to Newcastle-Otawa scale for non randomized trials and according to Jadad score for randomized control trials.

## Discussion

Indwelling catheter removal is considered one of the most important steps of the robotic radical prostatectomy that may significantly influence both functional outcomes and complications rates. Surgeons main concerns include anastomotic leakage with potentially devastating results, ureteral stricture, urinary retention requiring readmission or reoperation and of course the duration and severity of postoperative urinary incontinence. Although several studies support early catheter removal, many surgeons remain hesitant to adopt this practice and alter their standard post operative plan.

### Definition of early catheter removal

It does not seem to be a consensus about the definition of early removal. There are studies that report removal of the catheter as early as post operative day (POD) 1 (9, 10), others POD 2 (11–14) and others on POD 3–4 (15–18). The list of the available studies is completed with few surgeons that point the early removal on POD 7 or later (19, 20). Most studies compare early vs. late removal, while some report outcomes of early removal without any direct comparison with group of late removal. Based on the published data, catheter removal on POD 3–4 appears to be the safest and most representative timing for early removal.

### Preoperative patient's characteristics

Across all studies, groups were matched for key preoperative factors that may influence the final outcomes (BMI, PSA, Gleason Score, stage, prostate volume) with no statistically significant differences (10, 14, 18). Most patients were relatively young (57–65 years old), had a BMI of 25–27 kg/m^2^ and small to moderate prostate size (40–50 mL) (9–12, 14–20). The majority of included patients were operated for local or locally advanced cancer (T2 to T3a) with ISUP 1–3 and with no important lower urinary tract symptoms (IPSS 4–8) (11, 13–15).

The patients that were selected for inclusion in these studies represent the typical patient prostate cancer population that is commonly undergo robotic radical prostatectomy; therefore, and the reported outcomes are applicable to most urologic practices. We recommend that surgeons adopting early catheter removal begin with patients who have smaller prostate glands with low volume and organ confined disease. Baseline characteristics of patients are presented in Table 1.

### Perioperative and postoperative outcomes

Most studies did not emphasize perioperative outcomes focusing instead on complications and long-term outcomes after the early removal of catheter. Available data indicate no difference in estimated blood loss (EBL) (9, 11, 13, 15, 18) or operating time (12, 15, 17) between groups suggesting comparable surgical quality. However, the randomized control study (RCT) by Lista et al, reported a significantly shorter hospital stay for early removal (4 days vs. 6 days *p* < 0.001) (18). Importantly, the rate of anastomotic leakage (after catheter removal) was very low (0.9%–2%) (12, 14) and it does not seem to differ between early and standard of prolonged removal (9, 17, 18, 21). In our opinion this is one of the main outcomes (along with rates of continence, ureteral stricture or bladder neck contracture, retention and readmission) that can help to turn the tide towards early removal, and it seems to be in favor of early catheter removal. Despite the fact that the only data we have for readmission of patients comes from one study, the rate of readmission seems to be as low as 4.3% another factor that implies that early removal of the catheter is safe and efficient (12). The outcomes are shown in Table 2.

**Table 2:** Perioperative and postoperative characteristics of included patients.

Study	Catheter days (pod)	Primary endpoints	Outcomes	Complications
Harke et al. (2020)	5 vs. SPT 5 vs. SPT 2 (TUC 1)	Postoperative continence	Better continence: SPT2	no difference
Lista et al. (2018)	3 vs. 5	Acute urinary retention (AUR) and urinary leakage rate	AUR 1 case for each group	higher urethral pain & economic burden for 5 pod (*p* = 0,03)
Prasad et al. (2014)	7 vs. SPT 7 (TUC 1)	Postoperative pain	no difference in pain	no difference
Lenart et al. (2024)	<14 vs. >14	Subsequent surgeries, complications & functional outcomes	no increase in subsequent surgeries	higher anastomotic strictures for >14 pod
Paludo et al.	1	Postoperative pain & continence	81% immediate continence, 95,2% at 3 months	AUR 1 patient
Gratzke et al.	2 vs. 6	Spontaneous voiding after catheter removal	no difference between groups, higher Qmax 6 pod	no difference
Brasetti et al.	2	Postoperative continence	29% immediate continence, 67% & 92% 3 & 6 months	22 CD 2 & 2 CD 3B complications
Hao et al.	7 vs. 10 vs. ≥14	Postoperative continence & overactive bladder symptom score (OABSS)	7 pod better continence results and lower OABSS at 4 and 24 weeks after TUC	lower OABSS for 7 pod group
Taylor et al.	SPT 6 (TUC 1) vs. SPT 6 (only/no TUC)	Postoperative continence & complications	Higher pad-free rate in the SPT-only group (*P* = .04) at 3 months	no differences in anastomotic leak, ileus, or urethral stricture.
Lenart 2 et al. (2019)	4 vs. 7	Acute urinary retention & UTI	AUR rates higher for early removal (pod 4)	Catheter indwelling time not risk factor for UTI
Alnazari et al.	4 vs. 7	Acute urinary retention & Postoperative continence	AUR rates higher for early removal (pod 4) & earlier return of urinary continence in pod 4 patients experiencing AUR.	0,9% anastomotic leak, 0,4% bladder neck contracture (71% pod 4)
Develtere et al.	2	Postoperative continence & complications	early (3 mo) urinary continence rate 67% & median time to urinary continence recovery 1 mo. After median follow-up of 18 mo, 88% continent	13% AUR. No anastomotic strictures
Khemees al. (2013)	3–7	Acute urinary retention	catheter removed an average of 4.1 vs. 5.7 pod in patients with vs. without AUR	no difference in urine leak

SPT, suprapubic tube; TUC, transurethral catheter; POD, post operative day; CD Clavien-Dindo system; AUR, acute urinary retention; OABSS,overactive bladder system score.

### Functional outcomes

Key outcomes influenced by catheter removal timing include continence recovery, potency and of course the long-term ureteral stricture (or bladder neck contracture) formation. The data for continence are very revealing: continence recovery occurs sooner with early catheter removal, whereas prolonged catheterization may have a negative long-term impact (14, 20). Immediate continence rated after early removal seems to be as high as 86% and increases to 90% in 3rd postoperative month (9). These data are reinforced from the study of Hao et al, with a rate of 63% of immediate continence after POD 2 and only 46% for the standard POD 7 (19). Nevertheless these outcomes come with the cost of higher urinary retention rates which are reported at 11%–13% (12, 13) and with a clear disadvantage for the groups of early removal (1.5 vs. 9.5% *p* < 0.01) (17). In this point we must stress the fact that this complication does not have an impact on any long term outcomes, including urethral stricture or bladder neck contracture since all studies did not find any statistical significant difference between the analyzed patients (14, 16, 18, 20, 21).

### Pain scores and quality of life

Pain and discomfort from indewelling catheterization remain major postoperative complaints and are often cited as reasons for early removal. The main goal of a minimal invasive procedure is to decrease pain and discomfort of the patient and enable him to return to his everyday life more rapidly.

Although available data are limited, one study reported no statistical significant differences in pain scores (urethral, perineal and abdominal VAS scores) between POD 2 and POD 7 (13). A very interesting finding, however, comes from the study of Prasad et al. who compared patients with early catheter removal but with the presence of suprapubic catheter (SPT). Even though there were no important differences in pain and discomfort scales, when patients were asked about how bothersome is the (indwelling)) catheter, in POD 0 only 21% of the patients answered greatly (with 21% in the SPC group) whereas in POD7 the rate was 41% (vs. 25% in the SPC group). This directly implies that the discomfort is increasing according to the days of catheterization.

## Conclusions

Although current evidence remains limited, early urethral catheter removal after robotic radical prostatectomy appears to be both safe and feasible. Surgeons considering this approach should begin with patients with organ-confined disease, low to moderate prostate volume disease and counsel them regarding potential short-term disadvantages. With careful patient selection early removal can enhance the minimal invasive benefits of robotic prostatectomy.

## Data Availability

The original contributions presented in the study are included in the article/Supplementary Material, further inquiries can be directed to the corresponding author/s.
